# Thyroid Hormone Upregulates Cav1.2 Channels in Cardiac Cells via the Downregulation of the Channels’ β4 Subunit

**DOI:** 10.3390/ijms251910798

**Published:** 2024-10-08

**Authors:** Elba D. Carrillo, Juan A. Alvarado, Ascención Hernández, Ivonne Lezama, María C. García, Jorge A. Sánchez

**Affiliations:** Department of Pharmacology, Center for Research and Advanced Studies of the National Polytechnic Institute, Mexico City 07360, Mexico; elcarrillo@cinvestav.mx (E.D.C.); juanandres.alvarado@cinvestav.mx (J.A.A.); ashernandez@cinvestav.mx (A.H.); ivonne.lezama@cinvestav.mx (I.L.); cgarcia@cinvestav.mx (M.C.G.)

**Keywords:** Cav1.2 channels, β4 subunit, thyroid hormone, H9c2 cell line, pCREB

## Abstract

Thyroid hormone binds to specific nuclear receptors, regulating the expression of target genes, with major effects on cardiac function. Triiodothyronine (T3) increases the expression of key proteins related to calcium homeostasis, such as the sarcoplasmic reticulum calcium ATPase pump, but the detailed mechanism of gene regulation by T3 in cardiac voltage-gated calcium (Cav1.2) channels remains incompletely explored. Furthermore, the effects of T3 on Cav1.2 auxiliary subunits have not been investigated. We conducted quantitative reverse transcriptase polymerase chain reaction, Western blot, and immunofluorescence experiments in H9c2 cells derived from rat ventricular tissue, examining the effects of T3 on the expression of α1c, the principal subunit of Cav1.2 channels, and Cavβ4, an auxiliary Cav1.2 subunit that regulates gene expression. The translocation of phosphorylated cyclic adenosine monophosphate response element-binding protein (pCREB) by T3 was also examined. We found that T3 has opposite effects on these channel proteins, upregulating α1c and downregulating Cavβ4, and that it increases the nuclear translocation of pCREB while decreasing the translocation of Cavβ4. Finally, we found that overexpression of Cavβ4 represses the mRNA expression of α1c, suggesting that T3 upregulates the expression of the α1c subunit in response to a decrease in Cavβ4 subunit expression.

## 1. Introduction

The rhythmicity and contractility of the heart are regulated extensively. Thyroid hormones are known to increase heart rate and cardiac contractility, and their major effects on the heart are mediated by triiodothyronine (T3). T3 increases the force and speed of systolic contraction and the speed of diastolic relaxation [1]. It has genomic effects and regulates gene expression by binding with high affinity to T3 receptors that repress or activate the transcription of T3 targets through specific response elements [2]. Cardiac ion channels are targets of T3; specifically, the duration of the action potential in the atria is shortened with an increase in the amplitude of ultrarapid delayed rectifier K^+^ currents, following an increase in the expression of the mRNA of voltage-gated Kv1.5 channels [3].

Ca^2+^ ions play a crucial role in cardiac excitation-contraction coupling, a process that enables the chambers of the heart to contract and relax. Cardiac action potentials lead to an increase in [Ca^2+^]_i_, which is initiated by the opening of voltage-gated Cav1.2 channels, leading to a Ca^2+^ influx through the principal α1c subunit [4]. T3 reduces the expression of α1c in rat atria [3] and HL-1 cells [5], which have a gene expression pattern similar to that of adult atrial cardiac myocytes [6]. The downregulation of the α1c subunit by T3 is mediated by decreases in the phosphorylation and nuclear translocation of the transcription factor cyclic adenosine monophosphate response element-binding protein (CREB) [5]. However, no existing report describes the actions of T3 on other Cav1.2 channel subunits, and information on the actions of T3 on ventricular myocytes is limited.

In this study, we used H9c2 cells to examine the effects of T3 on the expression of the α1c subunit and Cavβ4, an auxiliary subunit of Cav1.2 channels that in neurons is translocated to the nucleus and interacts with thyroid hormone receptor-α, acting as a repressor recruiting platform to control neuronal gene expression [7]. In cardiac cells, the Cavβ4 subunit also regulates gene transcription, as demonstrated by overexpression and knockdown experiments performed with H9c2 cells [8]. The H9c2 cell line is derived from rat left ventricular tissue, and its biochemical, morphological, electrical, and hormonal signaling properties have been characterized [9]. We performed Western blot and quantitative reverse transcriptase polymerase chain reaction (qRT-PCR) experiments to quantify protein and mRNA transcript level changes induced by T3 and immunofluorescence experiments to characterize T3-induced changes in the localization of the Cavβ4 subunit and phosphorylated (p)CREB. Finally, by overexpressing the Cavβ4 subunit in H9c2 cells, we tested the hypothesis that it regulates the expression of the α1c subunit gene (CACNA1C). Our findings contribute to the notion that Cavβ_4_ plays a role in gene expression, specifically affecting the principal subunit of Cav1.2 channels, and that T3 downregulates the Cavβ4 subunit and promotes the nuclear translocation of pCREB, which may be responsible for the upregulation of Cav1.2 channels.

## 2. Results

### 2.1. T3 Increased the Expression of α1c at the mRNA and Protein Levels in H9c2 Cells

**Figure 1:**
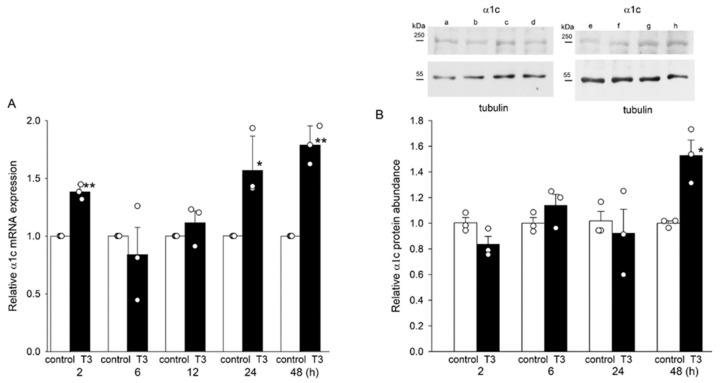
Relative mRNA expression and α1c protein abundance in H9c2 cells after incubation with T3. (**A**,**B**) Means ± SEMs of relative α1c subunit mRNA expression and protein abundance in H9c2 cells from three separate experiments. Insets show representative blots under control conditions (lanes a, c, e, and g) and 2, 6, 24, and 48 h after T3 treatment (lanes b, d, f, and h, respectively). Tubulin bands were used to normalize α1c subunit density values. In panels (**A**,**B**) each symbol represents a separate experiment. Original blots are presented in Figure S1. * *p* < 0.05. ** *p* < 0.01.

We found that the mRNA expression of the α1c subunit increased after 24 h treatment with T3 (Figure 1A). T3 also increased the protein abundance of the α1c subunit. Figure 1B shows representative blots of the α1c subunit and tubulin from cells incubated under control conditions and in T3 for 2, 6, 24, and 48 h. The increase in α1c protein abundance was seen after 48 h of T3 treatment (Figure 1B). Tubulin band densities were unchanged by T3 and were used for normalization.

### 2.2. T3 Decreased the Expression of Cavβ4 at the Protein Level and Its Nuclear Translocation in H9c2 Cells

**Figure 2:**
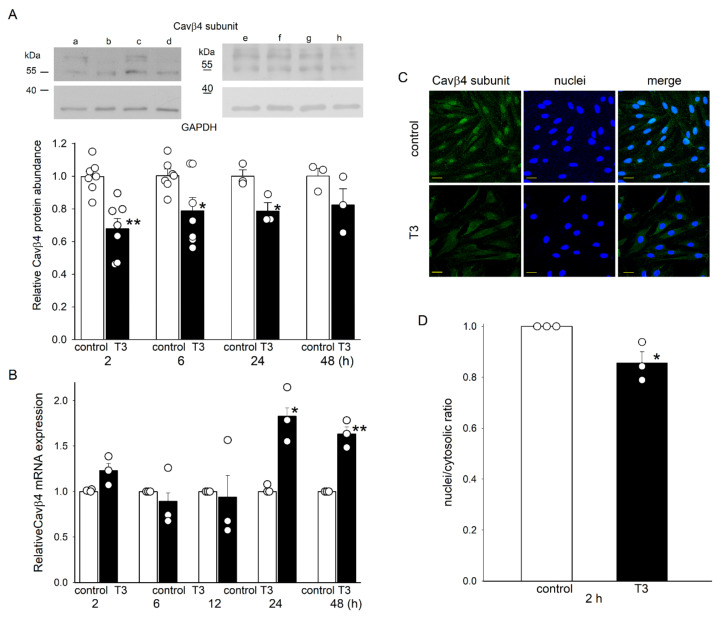
Relative Cavβ4 protein abundance in H9c2 cells after incubation with T3. (**A**) Means ± SEMs of relative Cavβ4 protein abundance in H9c2 cells (*n* = 3–7). Insets show representative blots under control conditions (a, c, e, and g) and 2, 6, 24, and 48 h after T3 incubation (lanes b, d, f, and h, respectively) from four separate experiments. GAPDH bands were used to normalize Cavβ4 subunit density values. Original blots are presented in Figure S1. (**B**) Means ± SEMs of relative Cavβ4 subunit mRNA expression in H9c2 cells after incubation with T3. (**C**) Confocal microscopic images of H9c2 cells under control conditions and after T3 treatment for 2 h. Representative images from three independent experiments show the co-localization of the Cavβ4 subunit (green) with Hoechst 33342-labeled nuclei (blue). Calibration bar, 32 μm. (**D**) Means ± SEMs of the nuclei/cytosolic ratio from three separate experiments. In panels (**A**,**B**,**D**), each symbol represents a separate experiment. * *p* < 0.05, ** *p* < 0.01.

T3 affected the expression of the Cavβ4 auxiliary subunit in a manner opposite to that observed for the α1c subunit. The antibody used recognized two protein bands (Figure 2A). The lower band had a molecular mass of slightly more than 55 kDa, as expected for the Cavβ4 subunit (which has a molecular mass of 58 kDa). The second band had a higher molecular weight, possibly reflecting the posttranslational modification of the Cavβ4 protein [8]. T3 rapidly reduced the integrated densities of the two Cavβ4 protein bands, whereas the GAPDH bands were unaffected and were used for normalization. The protein abundance of the two bands considered together decreased by more than 30% after 2 h incubation with T3. Thereafter, this reduction lessened (Figure 2A). The decrement in Cavβ4 protein abundance was not accompanied by a similar decrease in Cavβ4 mRNA expression, which remained unchanged at 2–12 h after T3 treatment and increased thereafter (Figure 2B). Consistent with the Western blot results, we found a decrease in Cavβ4 immunofluorescence in cells incubated in T3 for 2 h (Figure 2C). Interestingly, T3 treatment also reduced the nuclear/cytosolic Cavβ4 ratio (Figure 2D). The T3-induced reduction in the abundance of Cavβ4 protein was also observed in adult cardiomyocytes, though no changes in its nuclear/cytosolic ratio were observed (Figure S2).

### 2.3. Cavβ4 Overexpression Decreased α1c mRNA Expression and Protein Abundance in H9c2 Cells

**Figure 3:**
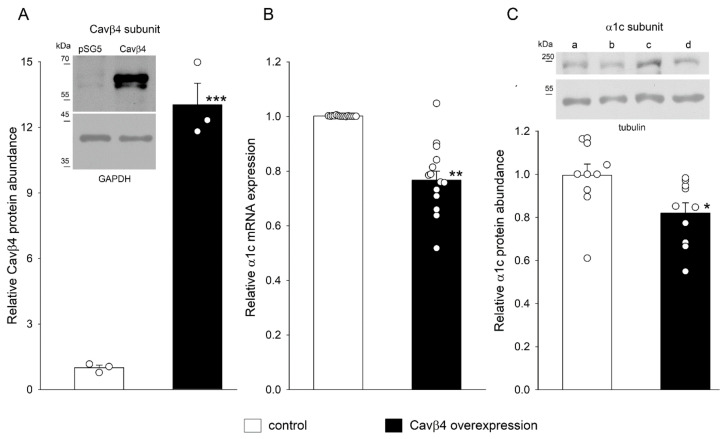
Relative α1c mRNA expression and protein abundance in H9c2 cells after Cavβ4 subunit overexpression. (**A**) Means ± SEMs of relative Cavβ4 protein abundance under control conditions and after Cavβ4 overexpression (*n* = 3). The inset shows representative blots of the Cavβ4 subunit and GAPDH bands; the latter was used to normalize Cavβ4 subunit density values. Original blots are presented in Figure S1. (**B**) Means ± SEMs of relative α1c subunit mRNA expression from control and Cavβ4 overexpression experiments (*n* = 14). (**C**) Means ± SEMs of α1c protein abundance from control and Cavβ4 overexpression experiments (*n* = 10). The inset shows representative blots of α1c subunit and tubulin bands under control conditions (lanes a and c) and after Cavβ4 overexpression (lanes b and d) from two separate experiments. Original blots are presented in Figure S1. In panels (**A**,**B**,**C**), each symbol represents a separate experiment. * *p* < 0.05, ** *p* < 0.01, *** *p* < 0.001.

The possibility that the decrease in the expression of the Cavβ4 subunit by T3 is involved in the upregulation of the α1c subunit was investigated next in Cavβ4 overexpression experiments. Figure 3A shows blots of Cavβ4 under control and Cavβ4 overexpression conditions. As expected, the density of the Cavβ4 bands increased markedly after Cavβ4 transfection. Overexpression of the Cavβ4 subunit resulted in decreased α1c mRNA expression and protein abundance, as shown in Figure 3B,C). The density of the α1c bands was reduced in β4-overexpressing cells, whereas the tubulin bands remained unchanged and were used for normalization.

### 2.4. T3 Altered the Nuclear Translocation, but Not the Protein Abundance of pCREB

**Figure 4:**
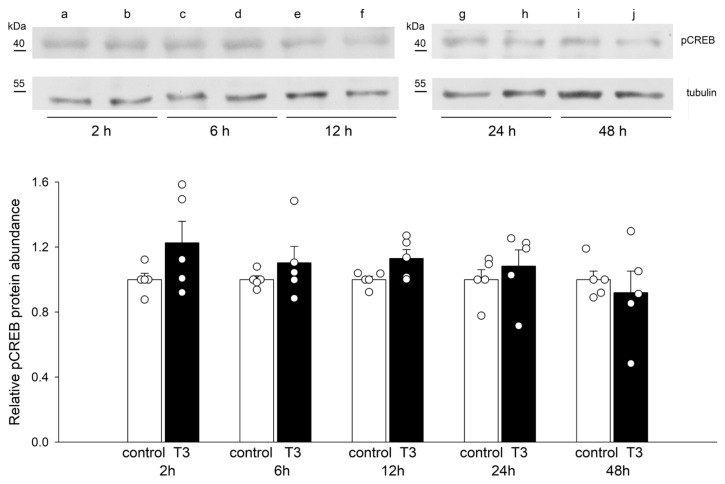
Relative abundance of pCREB in H9c2 cells after incubation with T3. Means ± SEMs of the relative abundance of pCREB under control conditions and after T3 treatment (*n* = 5). Each symbol represents a separate experiment. Representative blots of pCREB and tubulin under control conditions (lanes a, c, e, g, and i) and after T3 treatment for the indicated periods (lanes b, d, f, h, and j). Tubulin density values were used for normalization. Original blots are presented in Figure S1.

**Figure 5:**
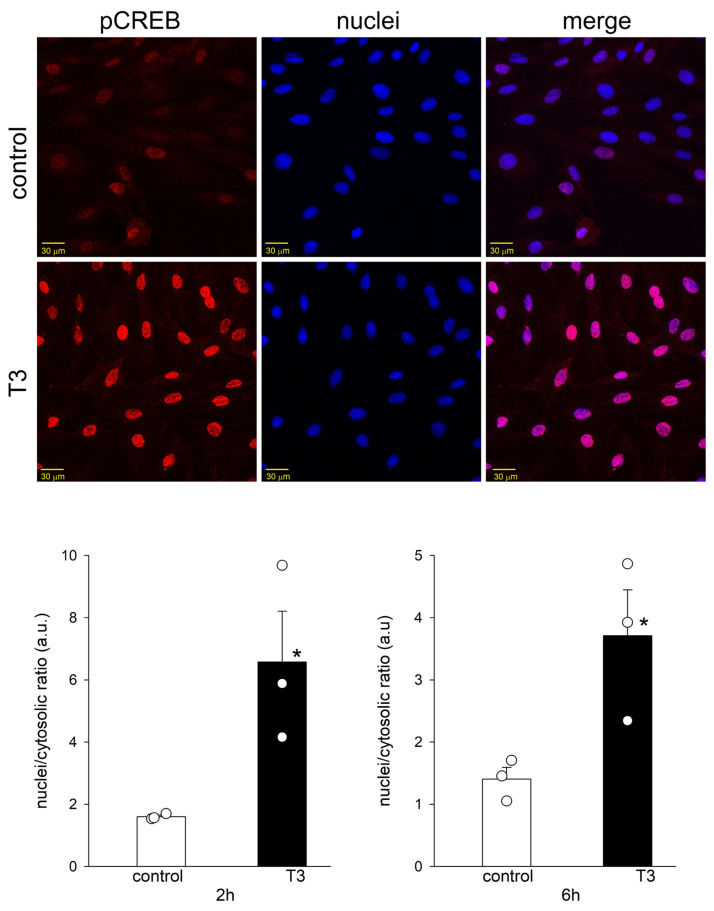
T3-induced translocation of pCREB to the nuclei of H9c2 cells. Confocal microscopic images of H9c2 cells under control conditions and after T3 treatment for 2 h. Representative images from three independent experiments show the co-localization of pCREB (red) with Hoechst 33342-labeled nuclei (blue). Calibration bar, 30 μm. Means ± SEMs of the nuclei/cytosolic ratio after T3 treatment for 2 and 6 h (*n* = 3). Each symbol represents a separate experiment. * *p* < 0.05.

Figure 4 shows blots of pCREB and tubulin from control and T3-treated H9c2 cells at 2, 6, 12, 24, and 48 h. No major change in the density of pCREB or tubulin bands was observed with up to 48 h incubation with T3. T3 treatment did not change the protein abundance of pCREB, but it induced the translocation of pCREB to the cell nuclei at 2 and 6 h (Figure 5). T3 also increased the nuclear translocation of pCREB in adult cardiomyocytes (Figure S2).

## 3. Discussion

In this study, we made the novel observation that T3 increases the mRNA expression and protein abundance of the α1c subunit of Cav1.2 channels. The expression of the α1c subunit mRNA had a distinct time course compared to that of its protein abundance. T3 produced an increase in the α1c subunit mRNA levels 2h after incubation and decreased thereafter, followed by another increase at 24 h. In contrast, α1c protein abundance was only observed after 48 h. There are precedents of a non-monotonic increase in mRNA expression by T3 of other mRNA targets [10]. In some cases, T3 changes the length of mRNA poly(A), altering its stability [11], and it can also modulate the translational rate of mRNA [12]. It is plausible that changes in the stability of α1c mRNA are responsible for the protein increase not following a monotonic time course. Differences in the time course of mRNA expression and protein abundance of Cav1.2 channels by T3 have also been observed in atria-derived cardiomyocytes; T3 produced an early and non-sustained decrease in α1c mRNA expression while a decrease in protein content was only observed after 24 h [5]. Our results in ventricle-derived H9c2 cells are contrary to these previous observations in HL-1 cells and may be related to the effects of T3 on the Cavβ4 subunit (see below).

We found that T3 also decreased Cavβ4 subunit protein abundance and translocation to the nuclei of H9c2 cells but did not decrease Cavβ4 mRNA expression. This last observation is not entirely surprising, as the abundance of many proteins is controlled by posttranscriptional phenomena in which microRNAs play leading roles [13]. For example, Cavβ2 subunit protein levels in cardiac muscle are reduced by microRNAs during long-term exposure to isoproterenol, while mRNA levels remain unchanged [14]. Cavβ4 protein abundance may also be regulated by microRNAs during T3 treatment; future research is needed to test this prediction directly. We further showed that T3 increased the nuclear translocation of the transcription factor pCREB and that Cavβ4 is a repressor of αlc subunit expression, suggesting that the upregulation of the α1c subunit by T3 is related to the downregulation of the Cavβ4 subunit. Increased nuclear translocation of pCREB after 2 h T3 treatment was also observed in adult ventricular cardiomyocytes, but Cavβ4 downregulation required longer T3 treatment in these cells than in H9c2 cells (Figure S2), preventing the exploration of possible changes in α1c subunit expression in adult tissue.

Cavβ subunits have been known for many years to play essential roles in the regulation of channel gating properties and in assisting the pore-forming α1 subunit to reach its required destination in the cell membrane [15]. More recently, Cavβ proteins have emerged as key players that regulate gene expression through DNA remodeling, modify transcription factor activity, and act as transcription factors themselves [16]. Four distinct genes encode Cavβ subunits, and all of them are expressed at the protein level in the ventricles of the adult heart [17]. The Cavβ4 subunit is localized in the nuclei and cytosol of H9c2 cells [8]. Its nuclear localization suggests that it plays a role in gene transcription, as demonstrated recently in neurons and cardiac cells [7,8].

The regulatory effects of Cavβ4 on gene expression depend on its interaction with transcription factors. In cardiac cells, Cavβ4 acts as an activator of interferon-related genes by interacting with interferon regulatory factor 7 [8]. In neurons, Cavβ4 acts as a repressor of the tyrosine hydroxylase (TH) gene by interacting with thyroid receptor-α (TR-α) [7,18]. TR-α plays an important role in the regulation of cardiac genes [19], and decreased contractile performance has been observed after null mutations of this receptor [20]. It is also expressed in H9c2 cells [21]. Cavβ4 can also repress gene expression by interacting with other nuclear proteins. For example, the cochlea exclusively expresses a short form of Cavβ4 that interacts directly with the chromo shadow domain of the nuclear chromobox protein 2 heterochromatin protein 1-γ, which is involved in gene silencing and transcription regulation [22]. Our results are consistent with the role of Cavβ4 as a transcriptional repressor of the αlc (CACNA1C) gene, as we observed a decrease in α1c mRNA expression in H9c2 cells when Cavβ4 was overexpressed.

The decrease in Cavβ4 protein expression induced by T3 was accompanied by an increase in mRNA expression and protein abundance of the α1c subunit. The upregulation of α1c mRNA is likely due to an increase in the transcription of CACNA1C, as we also observed that T3 induced the nuclear translocation of pCREB, the activated form of CREB [23]. pCREB regulates the α1c subunit mRNA’s expression [24,25]. This transcription factor binds to a cyclic adenosine monophosphate response element (CRE) upstream of the promoter of CACNA1C, increasing its gene promoter activity [26,27]. CREB knockout in cardiomyocytes leads to the reduction in L-type Ca^2+^ currents and mRNA α1c subunit levels [28]. Target genes of CREB include consensus sites for CREB binding in their promoter regions [23], and the CACNA1C promoter contains four CREB-binding regions [29].

Our results are consistent with previous findings regarding the effects of T3 on Ca^2+^ channels in ventricles. T3 augments transsarcolemmal Ca^2+^ influx in cultured ventricular myocytes and increases the number of L-type Ca^2+^ channels, as evidenced by [^3^H]-PN200-110 [30]. Consistent with these observations, a T3-induced increase in the L-type Ca^2+^ current magnitude has been observed in isolated ventricular myocytes [31,32], although non-genomic effects of T3 cannot be ruled out. On the other hand, fewer dihydropyridine binding sites have been observed in the ventricles of rats with hyperthyroidism relative to those of control animals [33]. The regulation of Cav1.2 channel expression is obviously more complex in whole animals than in cells; rats with hyperthyroidism develop cardiac hypertrophy, and decreases in the protein abundance of the α1c and Cavβ2 subunits due to proteasomal degradation have been observed in some models of hypertrophy [34].

**Figure 6:**
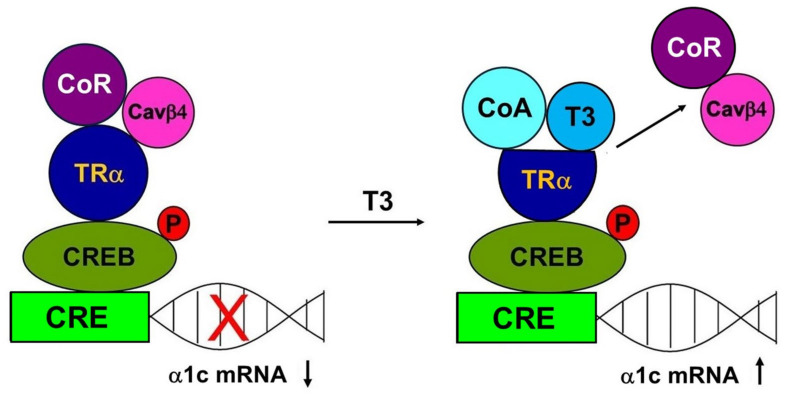
Role of Cavβ4 in pCREB regulation by T3. Schematic showing signaling mechanisms hypothetically involved in thyroid hormone actions on the CACNA1C gene via pCREB and the release of Cavβ4 inhibition of α1c subunit expression in ventricular cardiac cells.

In many cases, transcription regulation involves not only the binding of a transcription factor to regulatory sequences in target gene promoters but also transcriptional “cross-talk”. In this process, two transcription factors interact with each other in the promoter, but only one binds to it, usually resulting in the factors’ negative interference with each other’s activity [35]. Mutual transcriptional antagonism between TR and CREB through the factors’ direct association has been reported. Méndez-Pertuz et al. [36] reported that TR does not bind to CREs but is tethered to CRE-containing promoters when interacting with CREB in pituitary cells and noted that the association between TR and CREB inhibits CREB phosphorylation and represses the transcription of reporter genes [36]. This mechanism also appears to occur in atrial HL-1 myocytes [5]. No thyroid hormone response element in the promoter region of CACNA1C has been described, but T3 has an inhibitory effect on CREB phosphorylation and CACNA1C transcription, as evidenced by luciferase assays [5]. This repression is transient, as the decline in α1c subunit expression was observed between 0.5 and 2 h after incubation with T3 [5]. Cross-talk involving only two interacting transcription factors is likely an oversimplification of CACNA1C regulation by T3 in H9c2 cells; in addition to TR-α and CREB, Cavβ4 plays a role. Based on previous reports of protein-protein interaction between TR-α and Cavβ4 [7] and between TR and CREB [36], and on our observation of the repression of α1c subunit expression by Cavβ4, Cavβ4 is likely required for T3 repression of the CACNA1C gene. Our observation of the downregulation of the Cavβ4 subunit by T3 is also consistent with this scenario. Taken together, the previous and present data suggest that the Cavβ4 protein abundance shifts the balance between target gene repression and activation. In this regard, it is interesting to note that the distribution of Ca^2+^ channel subunits in the chambers of the heart is unequal, with Cavβ4 expressed more strongly in atria than in ventricles [37]. Hence, the repression of CACNA1C and downregulation of the α1c subunit by T3 prevails in atrial HL-1 myocytes, whereas T3 leads to the enhanced expression of the α1c subunit in H9c2 cells, as observed in the present study. We hypothesize that the Cavβ4 subunit acts as an associated protein to a corepressor (CoR) of CACNA1C gene expression (Figure 6). It has been established that, in the absence of T3, the unliganded TR recruits CoRs. Interaction of the unliganded TR with the complex of CoRs and its associated proteins leads to repression of transcription. The crystal structure of the rat TRα ligand-binding domain bound with T3 has revealed that T3 is part of the hydrophobic core. T3 establishes the active conformation of the receptor and induces structural changes, allowing the liganded TR to recruit coactivators (CoAs), leading to the dissociation of CoRs with a relief of repression and an association with CoAs that participate in the activation of transcription [38,39].

In conclusion, the main findings of this work are that Cavβ4 significantly reduces the mRNA expression of the principal α1c subunit of cardiac Cav1.2 channels and that T3 downregulates the expression of the auxiliary Cavβ4 subunit at the protein level and reduces its translocation to the nuclei of H9c2 cells. Additionally, we showed that T3 upregulates the α1c subunit at the mRNA and protein levels and increases the nuclear translocation of pCREB. These findings suggest that T3-induced α1c subunit upregulation may be explained in part by the decreased expression of the Cavβ4 subunit. Given the clinical importance of Cav1.2 channels, a detailed understanding of the cellular mechanisms underlying the regulation of Cav1.2 subunits is relevant to the development of cardioprotective therapies.

## 4. Materials and Methods

### 4.1. Animals

Male Wistar rats, 7–8 weeks old, weighing 250–300 g, were used in this study. Rats were obtained from the animal production and experimentation unit of the Center for Research and Advanced Studies of the National Polytechnic Institute (UPEAL-CINVESTAV-IPN; Mexico City, Mexico). They were housed in four per cage in a room maintained at a temperature between 21 and 24 °C with a 12:12 h light-dark cycle and fed standard rat chow and water ad libitum.

### 4.2. Heart and Ventricular Myocyte Isolation

We followed the heart and ventricular myocyte isolation methods described elsewhere [40]. Briefly, in preparation for heart extraction, each rat was anesthetized with 50 mg/kg sodium pentobarbital and given 500 U/kg heparin sodium solution, both via intraperitoneal injection. When the rat was completely unresponsive to stimulation, its heart was excised rapidly, arrested in Ca^2+^-free Tyrode’s solution (containing 136 mM NaCl, 5.4 mM KCl, 1 mM MgCl_2_, 10 mM HEPES, and 11 mM glucose), gassed with 95% O_2_/5% CO_2_ at pH 7.4, and perfused in a Langendorff apparatus with an aortic cannula for 5 min at 37 °C with Ca^2+^-free Tyrode’s solution. Chemicals were purchased from Sigma-Aldrich (Burlington, MA, USA). The heart was recirculated for 60 min with Ca^2+^-free Tyrode’s solution supplemented with 70 U/mL type II collagenase (Worthington, Lakewood, NJ, USA) and 0.5 mg/100 mL type XIV protease (Sigma-Aldrich, Burlington, MA, USA). The ventricles were minced and shaken two or three times at 45 rpm for 7 min in the same solution. The dislodged cells were filtered through a 100-μm nylon cell strainer (BD Falcon, Corning NY, USA) and centrifuged at 28× *g* for 2 min. The pellet was resuspended in Ca^2+^-free Tyrode’s solution with 1% bovine serum albumin (BSA, Sigma-Aldrich, Burlington, MA, USA). The [Ca^2+^]_o_ concentration was gradually increased to 1 mM.

### 4.3. Cell Culture and Transfection

We followed the general procedures for cell culture and transfection described elsewhere [8]. In brief, H9c2 cells (passages 17–24, American Type Culture Collection) were cultured in monolayers in Dulbecco’s modified Eagle’s medium (Gibco, Thermo Fisher Scientific, Waltham, MA, USA) supplemented with 10% fetal bovine serum (Gibco, Thermo Fisher Scientific, Waltham, MA, USA), sodium bicarbonate (1.5 g/L), penicillin (50 IU), and streptomycin (50 μg/mL) (Thermo Fisher, Waltham, MA, USA) under atmospheric conditions and 5% CO_2_ at 37 °C in a humidified incubator. Cells were used when they had reached 80–90% confluence, usually within 24–48 h. They were treated with or without T3 (T6397, 100 nmol/L; Sigma-Aldrich, Burlington, MA, USA) for the time periods shown in the figures. H9c2 cells were transiently transfected with a plasmid encoding the Cavβ4b subunit cloned by Castellano et al. [41] or an empty vector pSG5 plasmid with Lipofectamine 2000 (Invitrogen, Carlsbad, CA, USA) according to the manufacturer’s instructions. The medium was replaced after 4–6 h, and the cultures were maintained for a total of 48 h.

### 4.4. qRT-PCR Assays

Total RNA was isolated from H9c2 cells using the RNeasy mini kit (Qiagen, Germantown, MD, USA). Spectrophotometry (Implen NanoPhotometer, Munich, Germany) was performed for quantification. RT was performed with 500 ng deoxyribonuclease-treated RNA in 20-μL reactions. Complementary DNA was synthesized with Superscript III RT (Invitrogen, Carlsbad, CA, USA) and random hexamers (250 ng) according to the manufacturer’s instructions. To quantify mRNA, we used TaqMan assays (Applied Biosystems, Foster City, CA, USA) with an iCycler iQ (Bio-Rad, Hercules, CA, USA) using the TaqMan Gene Expression Master Mix (4369016) and the primer-probe sets for β4 (*Cacnb4*, Rn01449787_m1) and α1c (*Cacna1c*, Rn00709287_m1). Eukaryotic 18*S* ribosomal RNA (Hs99999901_s1) was used as an internal control. Quantification was performed using the 2^−ΔΔCT^ method [42].

### 4.5. Western Blotting

H9c2 cells were cultured in P100 plates and treated with control or T3 for predetermined periods. They were then scraped and placed in a lysis buffer [100 mM NaCl, 20 mM Tris-HCl (pH 7.5), and 1% Triton X-100]. Fresh cardiomyocytes were incubated in Tyrode’s solution with and without T3 for predetermined periods. Thereafter, myocytes were centrifuged at 500× *g* for 2 min and then homogenized in RIPA buffer containing 150 mM NaCl, 1% NP-40, 0.5% sodium deoxycholate, 0.1% sodium dodecyl sulfate (SDS), and 50 mM Tris-HCl (pH 8.0). Alternatively, myocytes and H9c2 cells were homogenized in a buffer containing 50 mM Tris HCl, 150 mM NaCl, 10 mM NaF, 1 mM Na_3_VO_3_, and 0.5% NP-40 (pH 7.4). The lysis buffers were supplemented with protease and phosphatase inhibitor cocktails (Halt™; Thermo Scientific, Waltham, MA, USA). The lysates were maintained on ice for 1 h and vortexed every 10 min. The samples were then centrifuged at 16,000× *g* for 15 min at 4 °C, and the supernatants were stored in liquid nitrogen until further use. Isolated protein contents were quantified using Bradford’s method [43]. Equal amounts of proteins were resolved by SDS-polyacrylamide gel electrophoresis, transferred to a nitrocellulose membrane (Bio-Rad, Hercules, CA, USA), and immunoblotted with appropriate antibodies. The membranes were blocked with 4.5% nonfat milk in phosphate-buffered saline (PBS) and incubated overnight at 4 °C with primary antibody. Thereafter, they were washed in PBS containing 0.1% Tween 20 and incubated in horseradish peroxidase (HRP)-conjugated secondary antibody for 1 h at room temperature. Chemiluminescence was detected with Immobilon western reagent (Millipore Co., Billerica, MA, USA). The antibody sources were polyclonal anti-rabbit α1c (ACC-003, 1:200; Alomone Labs, Jerusalem, Israel) and polyclonal anti-rabbit CACNB4 (1:500, 1:1000, A9304; ABclonal Technology, Woburn, MA, USA). We also used polyclonal anti-rabbit β-tubulin (1:5000, ab15568; Abcam, Waltham, MA, USA), monoclonal anti-rabbit pCREB (1:500, ab32096; Abcam, Waltham, MA, USA), monoclonal anti-mouse GAPDH (1:5000, G8795; Sigma-Aldrich, Burlington MA, USA), and HRP-conjugated anti-mouse or anti-rabbit (Invitrogen, Carlsbad, CA, USA) antibodies. The α1c, Cavβ4, and pCREB density values were normalized using GAPDH or tubulin bands.

### 4.6. Immunofluorescence

H9c2 cells were grown on coverslips in 24-well plates for confocal microscopic analysis. They were fixed with 4% paraformaldehyde, washed five times for 5 min each with PBS containing 1% BSA (Sigma-Aldrich, Burlington, MA, USA), and then permeabilized for 10 min with 1% Triton X-100 (Sigma-Aldrich, Burlington, MA, USA). Permeabilized cells were again washed five times with PBS and then blocked with 1% BSA and 1% Triton X-100 in PBS for 30 min at 4 °C. To detect Cavβ4, monoclonal anti-mouse CACNB4 (1:50, SMC-318; StressMarq, Biosciences Inc., Victoria, BC, Canada) and Alexa-Fluor 488 anti-mouse (1:200, A21202; Thermo Fisher (Waltham, MA, USA) secondary antibodies were used. To detect pCREB, anti-rabbit pCREB (1:100, ab32096; Abcam, Waltham, MA, USA) and anti-rabbit Alexa Fluor 555 (1:200, A31572; Thermo Fisher (Waltham, MA, USA) secondary antibodies were used. Permeabilized cells were incubated overnight with primary antibody at 4 °C, and the experimental procedures were then conducted at room temperature. Cells were subsequently washed five times with PBS for 5 min each and incubated for 1 h with a secondary antibody away from light. They were again washed five times with PBS-containing solution for 5 min each. Thereafter, the nuclei were counterstained with Hoechst 33,342 dye (1:1000, H3570; Thermo Fisher Scientific, Waltham, MA, USA) for 10 min and then washed five times with PBS for 5 min each to remove excess dye. Anti-fade fluorescence mounting medium (2 μL, Vectashield H1000; Vector Laboratories, Newark, CA, USA) was added to the center of each microscope slide, and the coverslip was gently transferred cell-side-down onto this surface. Primary and secondary antibodies were applied in PBS with 1% BSA.

Fresh cardiomyocytes were resuspended in normal Tyrode’s solution at room temperature and attached to laminin-coated coverslips for 2 h. Thereafter, they were washed five times with Tyrode’s solution, incubated in control or T3-containing solution for predetermined periods, washed five times in Tyrode’s solution, and fixed in 4% cold paraformaldehyde for 10 min at 4 °C. Fixed cardiomyocytes were then washed five times with PBS, blocked and permeabilized for 1 h with 0.3% Triton X-100 (Sigma-Aldrich, Burlington, MA, USA) and 5% Donkey serum (Invitrogen, Carlsbad, CA, USA) in PBS, and incubated overnight with primary antibody at 4 °C prepared in 0.5% BSA and 0.3% Triton X-100. Thereafter, they were washed five times with PBS and incubated for 1 h with a secondary antibody prepared in 0.5% BSA and 0.3% Triton X-100. The primary and secondary antibodies were the same as those used for the H9c2 cells. Cardiomyocytes were washed five times with PBS, incubated with Hoechst 33,342 nuclear dye (1:1000) for 10 min, washed again five times with PBS, and mounted on coverslips with 2 μL Vectashield (Vector Laboratories, Newark, CA, USA).

Confocal scanning microscopy was performed with argon (488 nm) and helium/neon (543 nm) lasers (TCS-SP8; Leica, Wetzlar, Germany). The lasers were used with an optimized pinhole diameter. Confocal images were obtained as z-stacks of single optical sections, then superimposed to create single images with the Leica LAS AF 2.6.0 build 7268 software. They were analyzed using the software ImageJ (NIH, Bethesda, MD, USA, ver. 2.7.0) [44].

To analyze the fluorescence ratio between nuclei and cytoplasm, we used image segmentation and mask-based calculations with Image J following the general procedure described elsewhere [45] with minor modifications. In brief, each image was color split, and the blue channel threshold was adjusted to select the nuclei; a binary image was then created, and these regions were saved (nuclei.roi). The color image of the fluorescence of interest was duplicated, and the threshold was adjusted to select the cells. The nuclei.roi file was then used to discard everything outside the nuclei, and the mean intensity value was obtained. The nuclei.roi file was also opened in the second image, and the nuclei area was then cleared, the remaining cytoplasm region was selected, and the mean intensity value was measured. The ratio was then obtained using the mean fluorescence values from the nuclei and from the cytoplasm.

### 4.7. Statistical Analysis

Data are expressed as means ± standard errors of the mean. The statistical analyses were performed using GraphPad Prism 4.0 (GraphPad Software Inc., Boston, MA, USA) and Sigma Stat 2.0 (Systat Software Inc., San Jose, CA, USA). For between-group comparisons, a Student’s *t*-test was used. For multiple comparisons of normally distributed data, a one-way analysis of variance followed by Dunn’s honestly significant difference test was performed. *p* values < 0.05 were considered to be significant.

## Data Availability

Data are contained within the article and Supplementary Materials.

## Supplementary Materials

The following supporting information can be downloaded at: https://www.mdpi.com/article/10.3390/ijms251910798/s1.
