# Supplementary material for: Thyroid Hormone Upregulates Cav1.2 Channels in Cardiac Cells via the Downregulation of the Channels’ β4 Subunit
Source: Int J Mol Sci. 2024 Oct 8;25(19):10798. doi: 10.3390/ijms251910798 (PMC11476369; doi:10.3390/ijms251910798)
Supplement: Supplementary file 1 [file ijms-25-10798-s001.zip › ijms-3207899-supplementary.pdf]

Supplementary results
(Supplementary Figures S1-S2)

Uncropped blot images for Carrillo *et al*. "**Thyroid hormone upregulates Cav1.2 channels in cardiac cells via the downregulation of the channels' β4 subunit**"

S1

Fig 1B

Nitrocellulose membranes, containing the protein bands were routinely cropped before hybridization. Membrane corners are indicated by arrows.

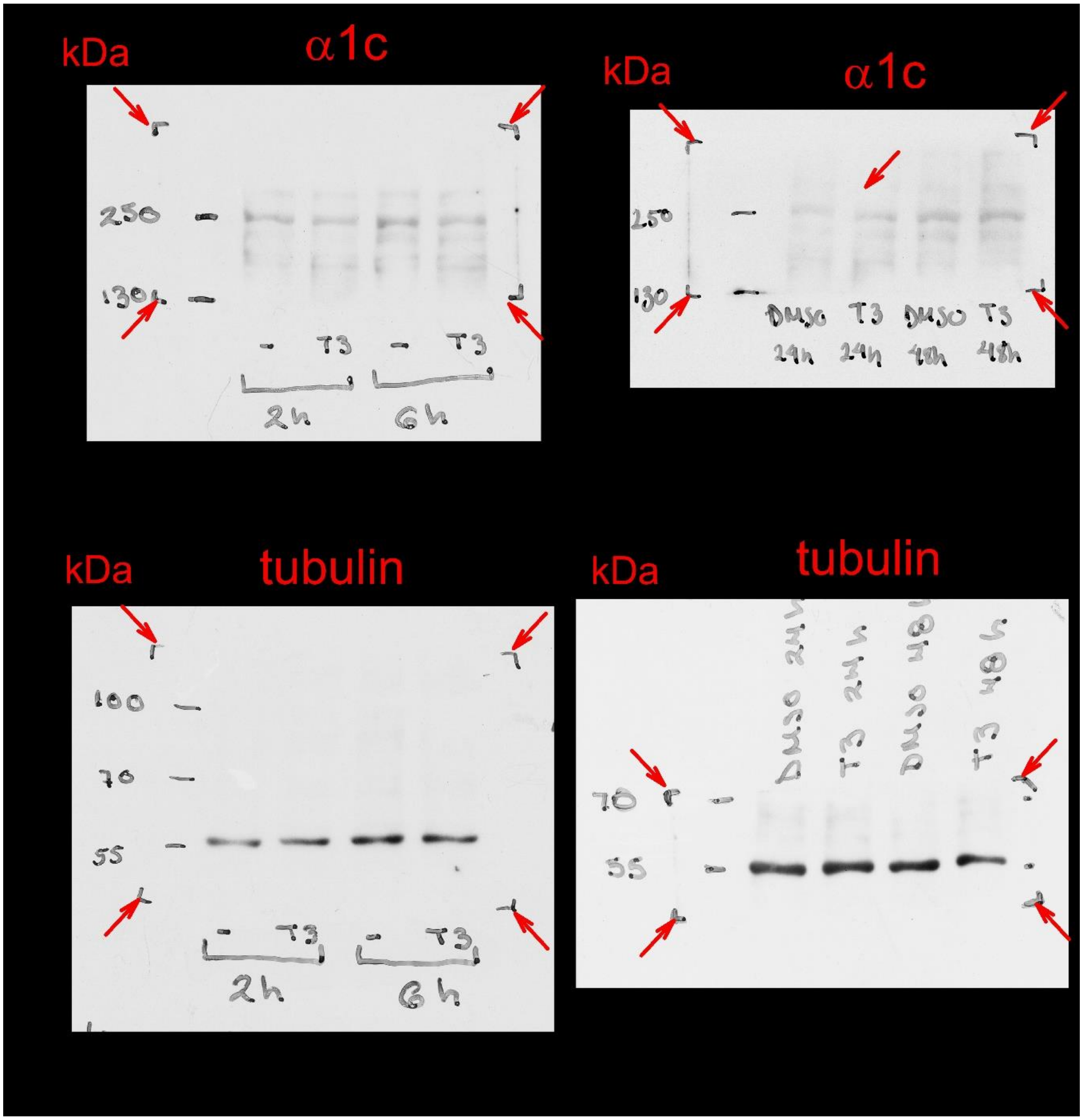

S2

Fig 2A

Nitrocellulose membranes, containing the protein bands were routinely cropped before hybridization. Membrane corners are indicated by arrows. Red boxes indicate areas of blots shown in the figure.

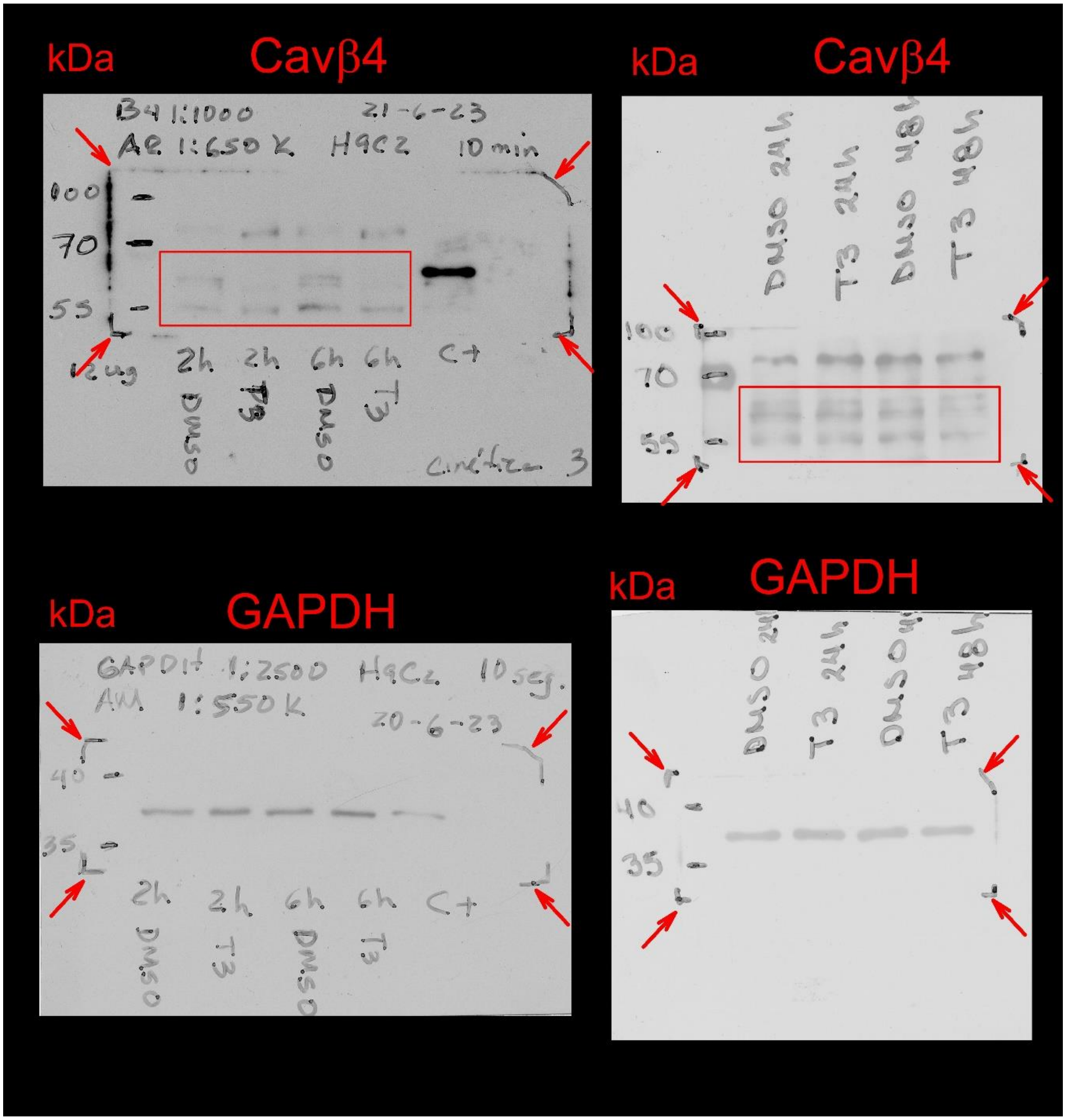

S3

Fig 3A

Nitrocellulose membranes, containing the protein bands were routinely cropped before hybridization. Membrane corners are indicated by arrows. Red boxes indicate areas of blots shown in the figure.

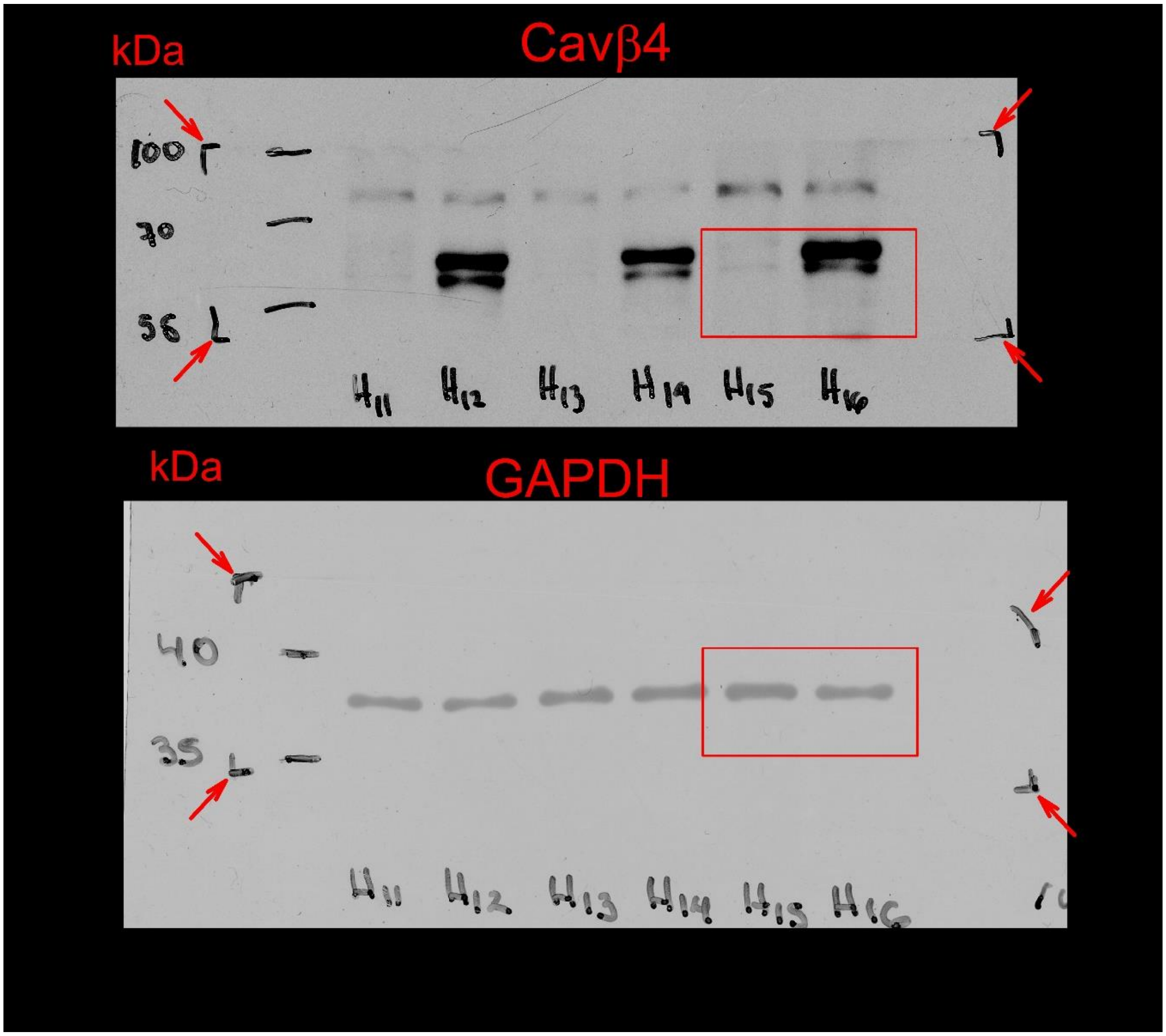

S4

Fig 3C

Nitrocellulose membranes, containing the protein bands were routinely cropped before hybridization. Membrane corners are indicated by arrows. Red boxes indicate areas of blots shown in the figure.

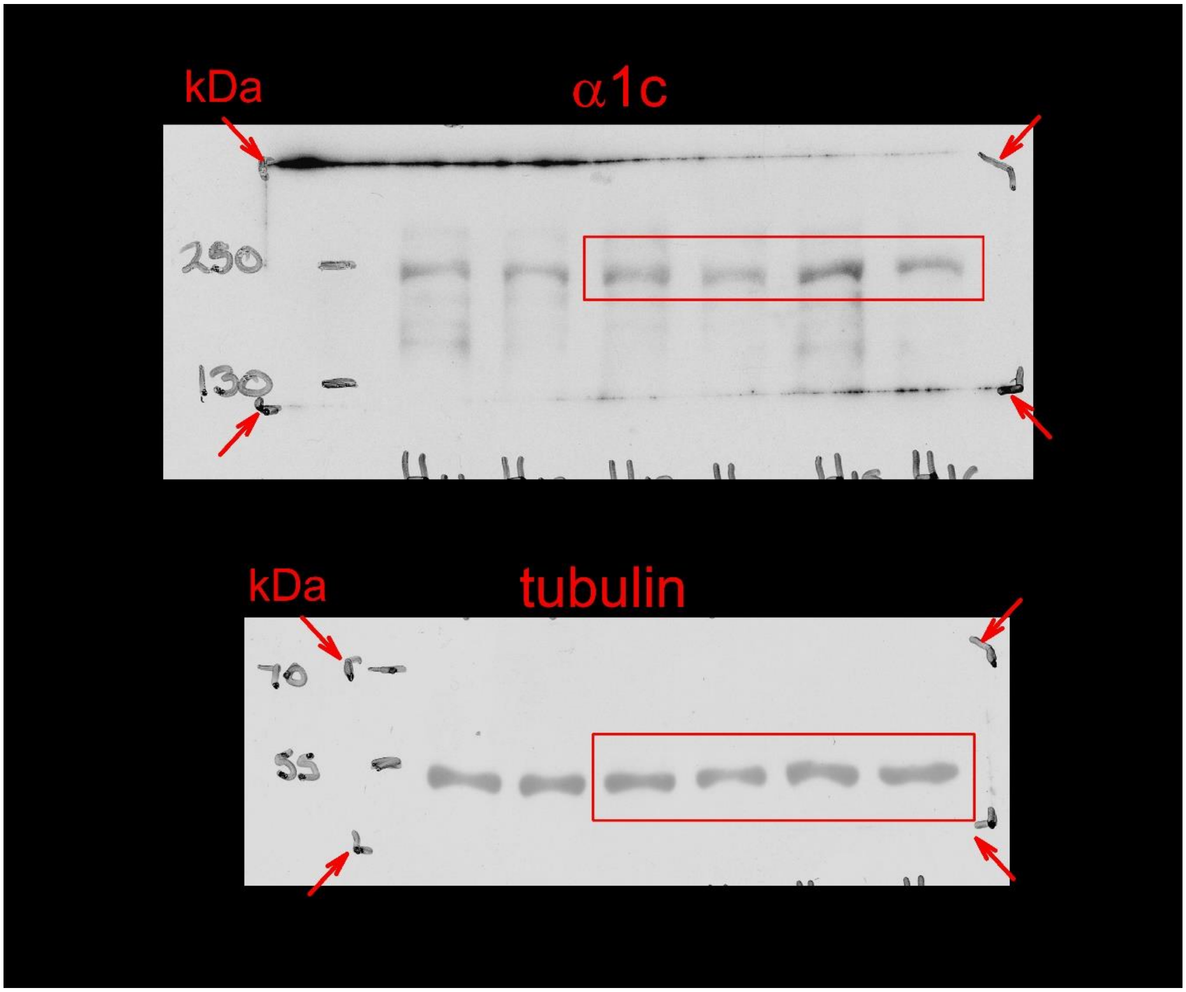

S5

Fig 4

Nitrocellulose membranes, containing the protein bands were routinely cropped before hybridization. Membrane corners are indicated by arrows.

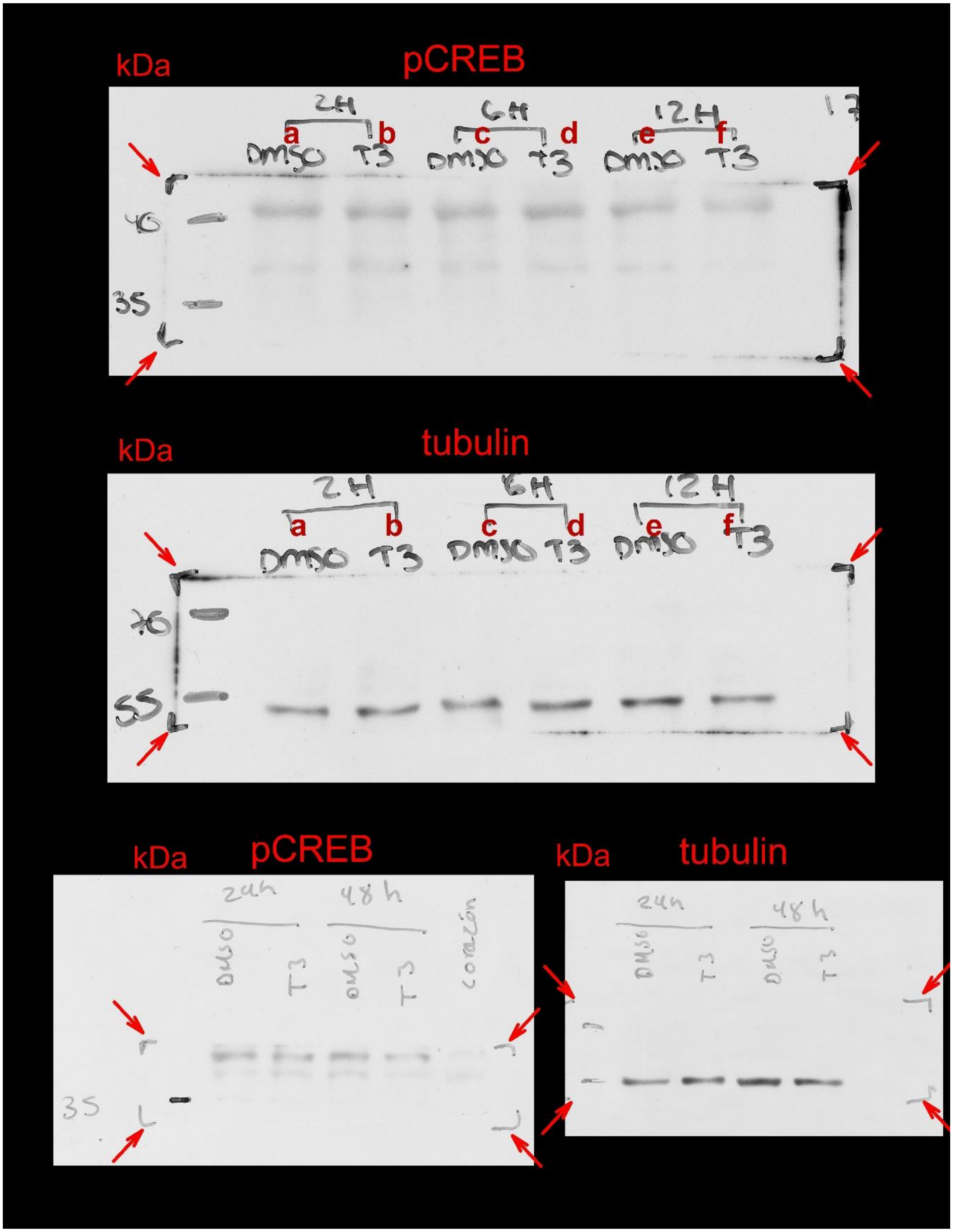

Fig S1A

Nitrocellulose membranes, containing the protein bands were routinely cropped before hybridization. Membrane corners are indicated by arrows.

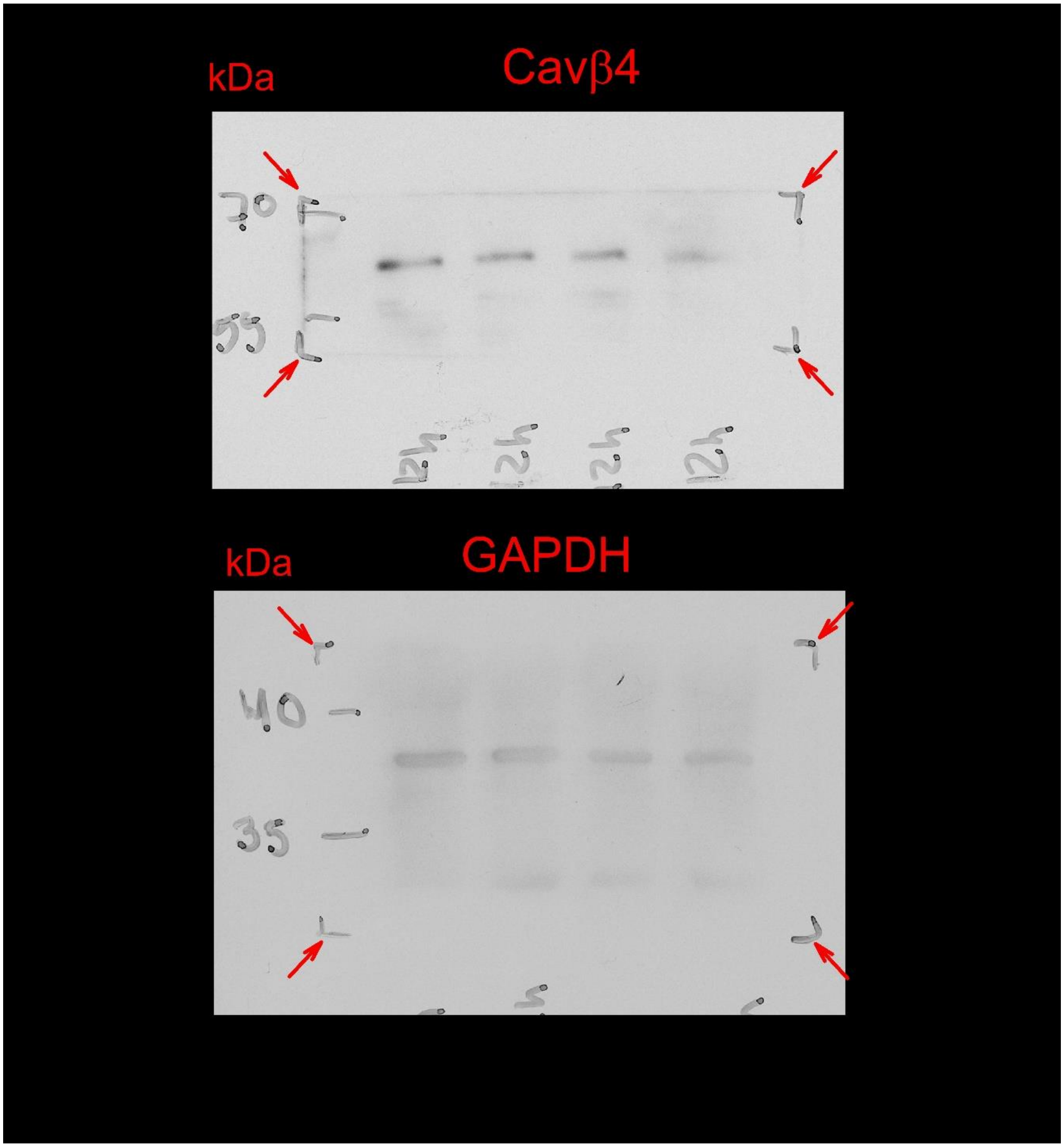

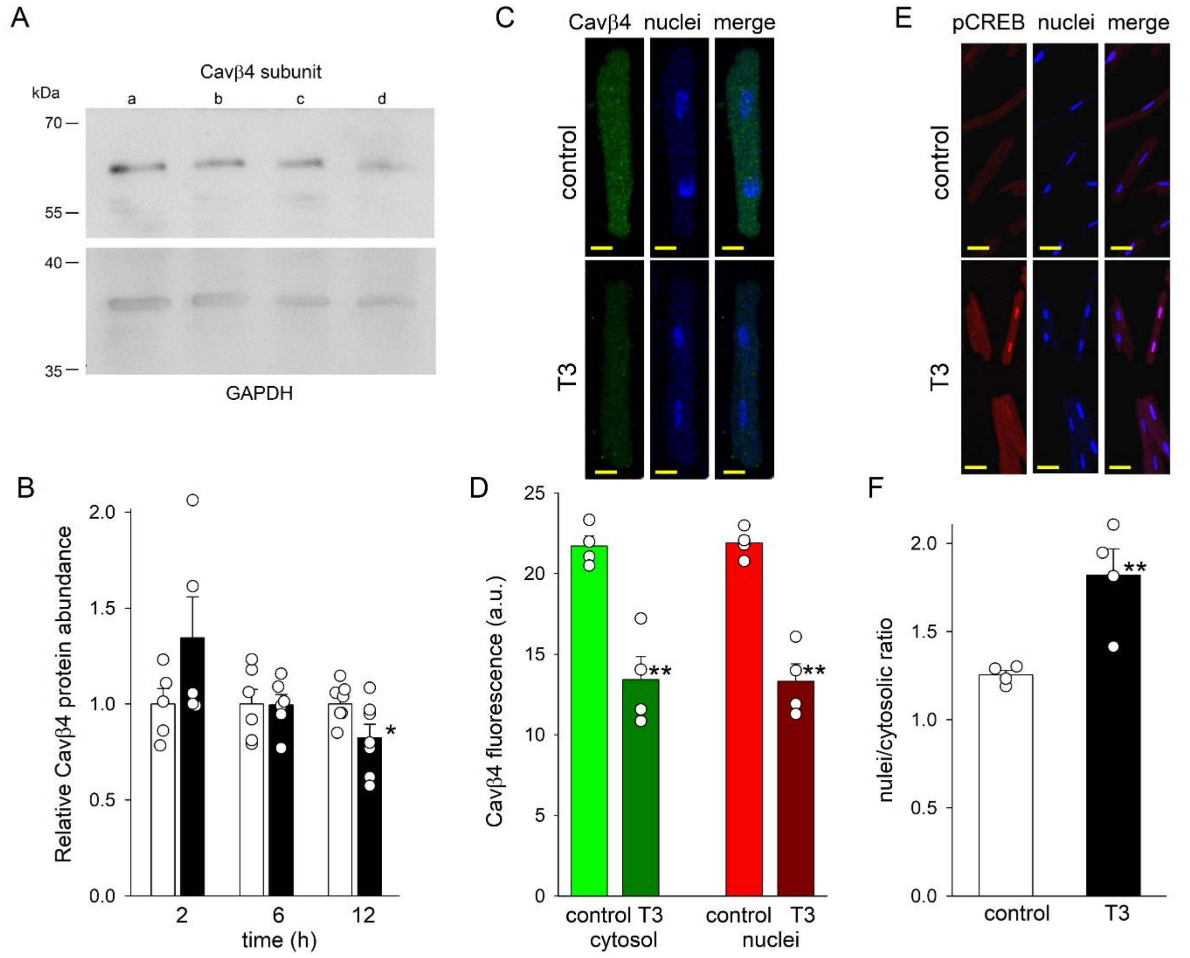

Figure S2. Effects of T3 treatment on the relative abundance of the Cavβ4 subunit and the nuclear translocation of pCREB in rat ventricular cardiomyocytes**.** (**A**) Representative blots of Cavβ4 subunit and GAPDH under control conditions (lanes a and c) and after 12 h T3 treatment (lanes b and d) from two separate experiments. Original blots are presented in Figure S2. (**B**) Means ± SEMs of the relative abundance of the Cavβ4 subunit under control conditions and after T3 treatment for the indicated periods (*n* = 7). Each symbol represents a separate experiment. GAPDH density values were used for normalization. (**C**) Representative images of cardiomyocyte immunofluorescence under control conditions and after T3 treatment for 24 h. Images show the co-localization of Cavβ4 (green) with Hoechst 33342–labeled nuclei (blue). Calibration bars, 14 mm. (**D**) Means ± SEMs of Cavβ4 fluorescence in the cytosol and nuclei after T3 treatment for 12–24 h (*n* = 4). Each symbol represents a separate experiment. Calibration bar, 14 mm. (**E**) Representative images of cardiomyocyte immunofluorescence under control conditions after 2 h T3 treatment. Images show the co-localization of pCREB (red) with Hoechst 33342–labeled nuclei (blue). Calibration bars, 30 µm. (**F**) Means ± SEMs of the nuclei/cytosolic ratios under control conditions and after 2 and 6 h T3 treatment (*n* = 4). Each symbol represents a separate experiment. *$p < 0.05$, **$p < 0.01$.
